# Is the variation in monocyte to high-density lipoprotein cholesterol ratio a predictor of major cardiovascular events after acute coronary syndrome?

**DOI:** 10.1590/1414-431X2022e12410

**Published:** 2023-01-09

**Authors:** E.T. Figueiredo, C.H. Miranda

**Affiliations:** 1 Divisão de Medicina de Emergência, Departamento de Clínica Médica, Faculdade de Medicina de Ribeirão Preto, Universidade de São Paulo, Ribeirão Preto, SP, Brasil

**Keywords:** Monocyte to high-density lipoprotein cholesterol ratio, Coronary artery disease, Acute myocardial infarction, Unstable angina, Major cardiovascular events

## Abstract

In clinical practice, we need to develop new tools to identify the residual cardiovascular risk after acute coronary syndrome (ACS). This study aimed to evaluate whether the monocyte to high-density lipoprotein cholesterol ratio (MHR) variation (ΔMHR) obtained during hospital admission (MHR1) and repeated in the first outpatient evaluation (MHR2) is a predictor of major adverse cardiovascular events (MACE) after ACS. One hundred ninety-one patients admitted for ACS were prospectively included. The ΔMHR was calculated by subtracting MHR1 from MHR2. Patients were followed for 166±38 days in which the occurrence of MACE was observed. The best cutoff for ΔMHR was zero (0), and individuals were divided into two groups: ΔMHR<0 (n=113) and ΔMHR≥0 (n=78). The presence of MACE was higher in the ΔMHR≥0 (22%) than in the ΔMHR<0 (7%), with a hazard ratio (HR) of 3.96 (95% confidence interval [CI]: 1.74-8.99; P=0.0004). After adjusting for confounders, ΔMHR≥0 remained an independent MACE predictor with an adjusted HR of 3.13 (95%CI: 1.35-7.26, P=0.008). In conclusion, our study showed that ΔMHR was an independent MACE predictor after ACS. Thus, ΔMHR is a potential marker of residual cardiovascular risk after ACS.

## Introduction

Acute coronary syndrome (ACS) is one of the leading causes of morbidity and mortality in developed and developing countries (1). Those who have already suffered an acute myocardial infarction (AMI) have a four to six times greater chance of a new event (2). Despite all available therapeutic resources, especially the high-potency statins, the reduction of the relative risk of a new acute event is generally between 25 and 35% (3). High-density lipoprotein cholesterol (HDL) levels are positively correlated with the thickness of the fibrous cap of the culprit lesion in ACS patients (4). Furthermore, these levels have a strong inverse correlation with inflammatory marker levels such as high-sensitivity C-reactive protein (hsCRP), interleukin-6 (IL-6), and tumor necrosis factor-alpha (TNF-α) (5). On the other hand, monocytes play a crucial role in atherosclerosis pathophysiology (6). In addition to being the precursors of foam cells (7), they are the primary source of several pro-inflammatory and pro-oxidant factors, leading to thrombosis and endothelial dysfunction (8). The expression of genes involved in atherosclerosis is as low as the serum monocyte count (9). The monocyte to high-density lipoprotein cholesterol ratio (MHR) ratio has been reported to predict major adverse cardiovascular events (MACE), especially after ST-segment elevation AMI (10-14). However, these previous studies only evaluated MHR obtained during hospitalization. We hypothesized that these patients have a higher MHR during ACS hospitalization, which usually decreases over time. The purpose of this study was to assess whether the variation in the MHR (ΔMHR) obtained during hospital admission (MHR1) and repeated in the first outpatient evaluation (MHR2) is an independent MACE predictor after ACS.

## Material and Methods

### Patient enrollment

The present study was conducted in the Coronary Care Units (CCU) and specialized Outpatient Center of the Clinics Hospital of Ribeirão Preto School of Medicine, São Paulo University. Patients admitted with ACS diagnosis were prospectively included in this study. The inclusion criteria were:

- Age ≥18 years old;
- Both genders;
- Definitive diagnosis of ACS including acute myocardial infarction (AMI) in patients presenting with ST-segment elevation, AMI in patients without persistent ST-segment elevation and unstable angina;
- Lipid profile and blood count collected during hospital admission;
- Outpatient clinical evaluation within four months after hospital discharge;
- Lipid profile and blood count collected during first outpatient evaluation;
- Adherence to high-potency statin treatment as standardized in the institutional protocol (atorvastatin 40-80 mg/day or rosuvastatin 20-40 mg/day).

The exclusion criteria were: active infection during hospital admission or outpatient evaluation, hematological disease, cancer disease, rheumatic disease, chronic kidney failure on dialysis, liver failure, severe valve disease, aortic dissection, in-hospital death during hospitalization due to ACS, and occurrence of MACE between hospital admission and the first outpatient evaluation.

This study was approved by the Research Ethics Committee of Ribeirão Preto School of Medicine, São Paulo University (protocol number 2.974.303) and followed the Declaration of Helsinki. The subjects or their relatives gave written informed consent before enrollment in this study.

### Clinical characteristics

We collected the demographic and clinical data from electronic medical records. Clinical diagnosis was based on current guidelines. Patients who smoked or stopped smoking in the last year were considered smokers. Left ventricle ejection fraction (LVEF) was obtained through two-dimensional echocardiography performed by experienced doctors. We also collected data about the medications during hospital discharge and outpatient evaluation. At the hospital discharge time, all patients and guardians received verbal guidance and a document with an outpatient evaluation and a new laboratory test scheduled around sixty days after hospital discharge. According to institutional protocol, all patients received a medical prescription following the current recommendations, and the standardized statin was atorvastatin 40-80 mg daily.

### Laboratory tests

Lipid profile of total cholesterol, high-density lipoprotein cholesterol (HDL), low-density lipoprotein cholesterol (LDL), and triglycerides were determined within the first 24 h of hospital admission through Atellica Solution Immunoassay (Siemens, Germany) and without fasting. Monocyte count was performed during hospital admission through an automatic hematology analyzer (Penta XL 80, Horiba, Japan). The first ratio between the number of monocytes and the value of HDL-cholesterol (MHR1) was calculated. The second blood sample was collected during the first outpatient evaluation after discharge, using the same methods for measurement, and the second ratio between the number of monocytes and the value of HDL-cholesterol (MHR2) was calculated. The MHR variation (ΔMHR) was calculated for all patients with the formula: ΔMHR = MHR2 - MHR1.

### Procedures performed during hospitalization

Our institution is a high-volume tertiary cardiology center with 24/7 percutaneous coronary intervention (PCI) facilities and experienced interventional cardiology staff. The initial ACS treatment was carried out following national and international recommendations. The indication for PCI and the technical aspects of these procedures, such as size and type of diagnostic catheter or guide, direct stent or pre-dilation, device selection, thrombus aspiration, and other adjuvant pharmacotherapy (including glycoprotein IIbIIIa receptor antagonists) were performed at the discretion of the interventional cardiology team. Most of the patients received bare-metal stents. Myocardial revascularization surgery (coronary artery bypass graft, CABG) was performed with standard surgical techniques. Complete revascularization of severe coronary stenosis through PCI or CABG was performed when possible.

### Follow-up and outcomes

Prospective follow-up was carried out by telephone contacts at 30, 60, 90, and 180 days after the first outpatient clinical evaluation. In addition, a review of the electronic medical record or telephone contact with the assistant physicians was carried out when necessary.

The primary outcome in this study was major adverse cardiovascular events (MACE) including cardiovascular mortality, new acute myocardial infarction, new ischemic stroke, new unplanned coronary revascularization, and hospitalization for heart failure. The secondary outcome analyzed was the all-cause mortality rate. These outcomes were defined according to the standardized definitions for cardiovascular and stroke endpoint events in clinical trials (15) and the fourth universal definition of myocardial infarction (16).

### Statistical analysis

The Freedman method was used to estimate the sample size. We considered that a lack of decrease in the ΔMHR would be associated with a 50% higher MACE occurrence during follow-up (hazard ratio of 1.5), a proportion of 70% of the patients with decreased MHR during follow-up, a power of 80%, and a significance level of 5%. Based on these parameters, we would need to include 198 patients (approximately 138 patients with decreased MHR and 60 patients with unaltered or increased MHR).

The Shapiro-Wilk test was used to assess the type of distribution of variables. Continuous variables with normal distribution are reported as means±SD and those with another type of distribution are reported as the median and interquartile range (IQR). The unpaired Student’s *t*-test was used to compare two continuous variables when they presented a normal distribution, and the non-parametric Mann-Whitney test was used for other types of distribution. The Wilcoxon matched-pair signed rank test was used to compare paired continuous variables. Categorical variables are reported as frequencies and percentages and were compared using the chi-squared test.

We analyzed MACE outcomes as a categorical variable (percentage of patients that had MACE on the 180th day after inclusion) and time-to-event was analyzed with the Kaplan-Meier curve and log-rank test. We calculated the hazard ratio (HR) and its respective 95% confidence interval (CI) to determine the MACE predictors. Multivariate analysis was performed using a Cox proportional hazard model, in which we included the variables that achieved a P-value <0.10 in the univariate analysis. We used the area under the receiver operating characteristic curve (ROC) (AUC) and its 95%CI to evaluate the MHR accuracy for MACE prediction. The STATA 13.1 software (USA) was used for statistical analysis and graph construction. We considered a two-tailed P-value less than 0.05 as statistically significant.

## Results

**Figure 1:**
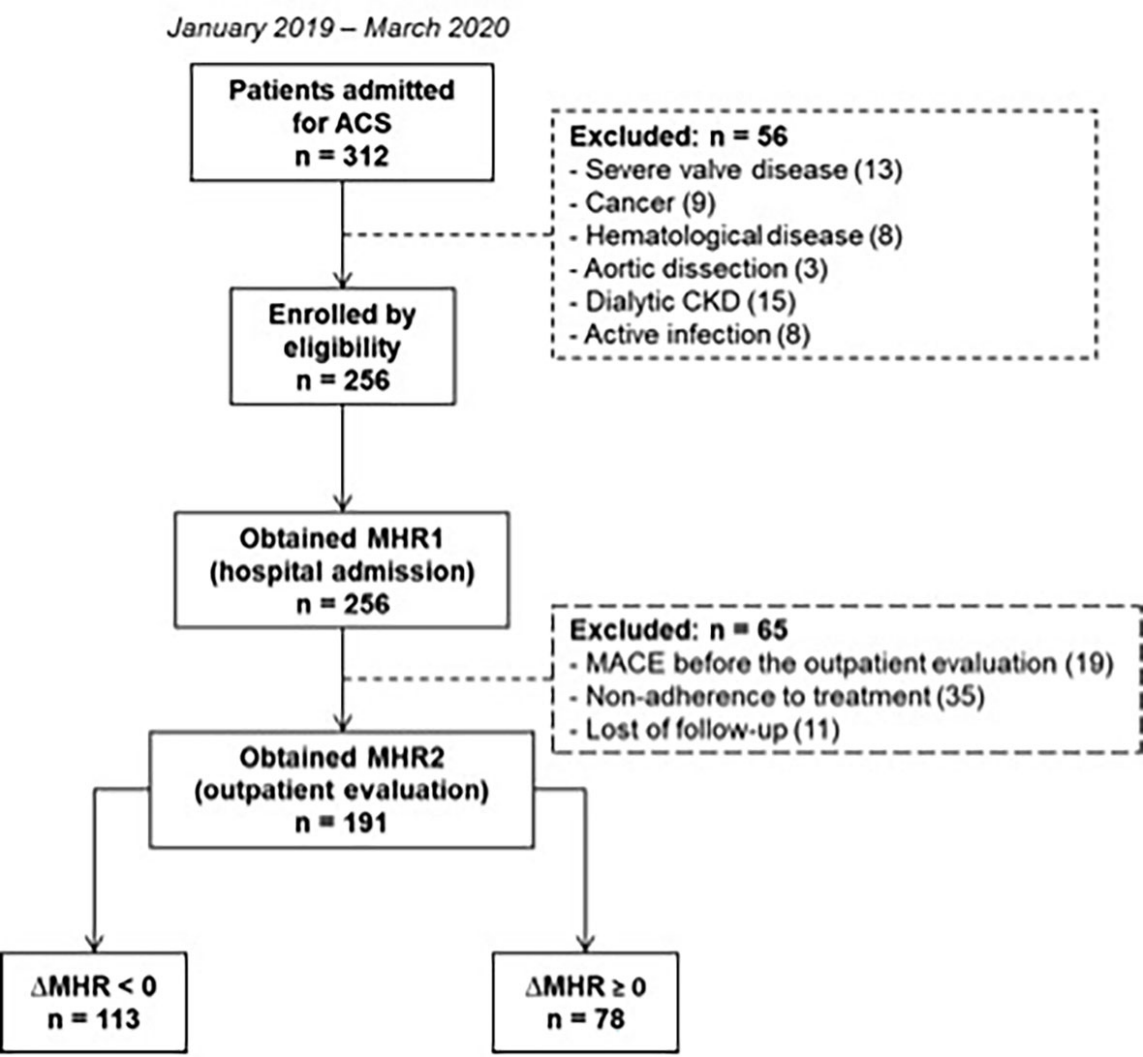
Flowchart of the patients included in this study. ACS: acute coronary syndrome; CKD: chronic kidney disease; MHR1: monocyte to high-density lipoprotein cholesterol ratio at hospital admission; MHR2: monocyte to high-density lipoprotein cholesterol ratio at first outpatient evaluation; ΔMHR: MHR2-MHR1.

Three hundred and twelve patients were hospitalized due to ACS between January 2019 and March 2020. Fifty-six patients were excluded during hospitalization according to the exclusion criteria. The MHR1 ratio was obtained in 256 patients. Then, other 65 patients were excluded during the outpatient evaluation. The MHR2 was obtained in 191 patients whose ΔMHR was also calculated (Figure 1).

Through the Youden index, the ΔMHR best cutoff point to determine the MACE occurrence was defined as 0.36. For practical reasons, we decided to approximate this cutoff value to 0 (zero). This cutoff value showed a sensitivity of 71% (95%CI: 51-87) and a specificity of 64% (95%CI: 57-72).

The patients were divided into two groups: one in which the ΔMHR decreased (ΔMHR<0) with 113 patients and another in which ΔMHR increased or was unaltered (ΔMHR≥0) with 78 patients. The baseline characteristics of patients in these two groups are exhibited in Table 1. The groups had no statistically significant differences in the demographic characteristics, cardiovascular risk factors, ACS types, coronary artery disease severity, coronary interventions, and medications during hospitalization or outpatient evaluation. The prognostic GRACE 2.0 score was similar between the groups: 113 (IQR: 93-130) *vs* 121 (IQR: 100-140), P=0.100. In the laboratory tests, only the monocyte count was statistically different between the groups: 500 cells/mm^3^ (IQR: 400-700) *vs* 400 cells/mm^3^ (IQR: 300-500); P=0.0001.

**Table 1:** Baseline characteristics of the patients according to the ΔMHR.

Characteristics	ΔMHR<0 (n=113)	ΔMHR≥0 (n=78)	P
Males	73 (64)	47 (60)	0.541
Age, years	62±11	62±13	0.966
Risk factors			
Hypertension	84 (74)	61 (78)	0.539
Diabetes	45 (39)	32 (41)	0.868
Dyslipidemia	36 (31)	22 (28)	0.589
Smoking	44 (38)	34 (43)	0.520
Prior AMI	33 (29)	26 (33)	0.544
Stroke	7 (6)	4 (5)	0.756
Prior CABG	7 (6)	7 (8)	0.469
Prior PCI	20 (17)	17 (21)	0.481
COPD	4 (3)	2 (2)	0.704
Heart failure	17 (15)	17 (21)	0.231
Atrial fibrillation	9 (7)	6 (7)	0.945
CKD	21 (18)	14 (17)	0.911
Length of hospital stay, days	4 (3−7)	5 (4−9)	0.216
Risk scores			
GRACE 2.0	113 (93−130)	121 (100−140)	0.100
GRACE 2.0 ≥140 points	20 (18)	21 (27)	0.127
SAPS III	35 (32−39)	36 (32−42)	0.143
ACS classification			0.269
Unstable angina	19 (16)	14 (17)	
Non-ST-elevation AMI	30 (26)	13 (16)	
ST-elevation AMI	64 (56)	51 (65)	
Killip classification			0.368
I	92 (81)	56 (71)	
II	13 (11)	12 (15)	
III	2 (1)	4 (5)	
IV	6 (5)	6 (7)	
Cardiopulmonary arrest	4 (3)	7 (8)	0.368
SBP, mmHg	123 (107−133)	120 (108−140)	0.996
HR, bpm	78 (69−90)	78 (69−87)	0.887
LVEF, %	52 (43−58)	53 (42−57)	0.600
Coronary angiography			0.256
No coronary lesions	5 (4)	0 (0)	
Single-vessel	33 (29)	27 (34)	
Two-vessel	34 (30)	25 (32)	
Three-vessel	41 (36)	26 (33)	
Laboratory tests			
Creatinine, mg/dL	1.08 (0.92−1.30)	1.08 (0.85−1.24)	0.694
Hemoglobin, g/dL	13.7 (12.4−15.5)	13.8 (12.0−15.3)	0.608
Blood glucose, mg/dL	115 (102−160)	123 (99−177)	0.956
LDL-cholesterol, mg/dL	103 (76−128)	96 (73−126)	0.646
HDL-cholesterol, mg/dL	34 (30−39)	37 (30−41)	0.078
Triglycerides, mg/dL	124 (93−179)	126 (94−170)	0.819
Monocytes, count per mm^3^	500 (400−700)	400 (300−500)	0.000
Medication at hospital discharge			
Aspirin	113 (100)	78 (100)	1.000
P2Y12 inhibitors	110 (97)	76 (97)	0.969
β blocker	99 (87)	64 (82)	0.286
ACEi	82 (72)	50 (64)	0.213
ARB	23 (20)	19 (24)	0.511
Calcium channel blocker	19 (16)	17 (21)	0.387
Nitrates	36 (31)	27 (34)	0.690
Statin	112 (99)	76 (97)	0.359
Warfarin	12 (10)	8 (10)	0.936
DOACs	5 (4)	3 (3)	0.844
Interventions			
Coronary angioplasty	73 (64)	58 (74)	0.153
Thrombolytic	7 (6)	7 (8)	0.469
CABG	2 (1)	5 (6)	0.093

Data are reported as n (%), mean±SD, or median (interquartile range). Mann-Whitney test, Student’s *t*-test, or chi-squared test were used for statistical analyses. MHR: monocyte to high-density lipoprotein cholesterol ratio; AMI: acute myocardial infarction; CABG: coronary artery bypass graft; PCI: percutaneous coronary intervention; COPD: chronic obstructive pulmonary disease; CKD: chronic kidney disease; GRACE: global registry of acute coronary events; SAPS: simplified acute physiology score; ACS: acute coronary syndrome; SBP: systolic blood pressure; HR: heart rate; LVEF: left ventricle ejection fraction; LDL-cholesterol: low-density lipoprotein cholesterol, HDL-cholesterol: high-density lipoprotein cholesterol; ACEi: angiotensin converting enzyme inhibitors; ARB: angiotensin receptor blockers; DOACs: direct-acting oral anticoagulants.

**Figure 2:**
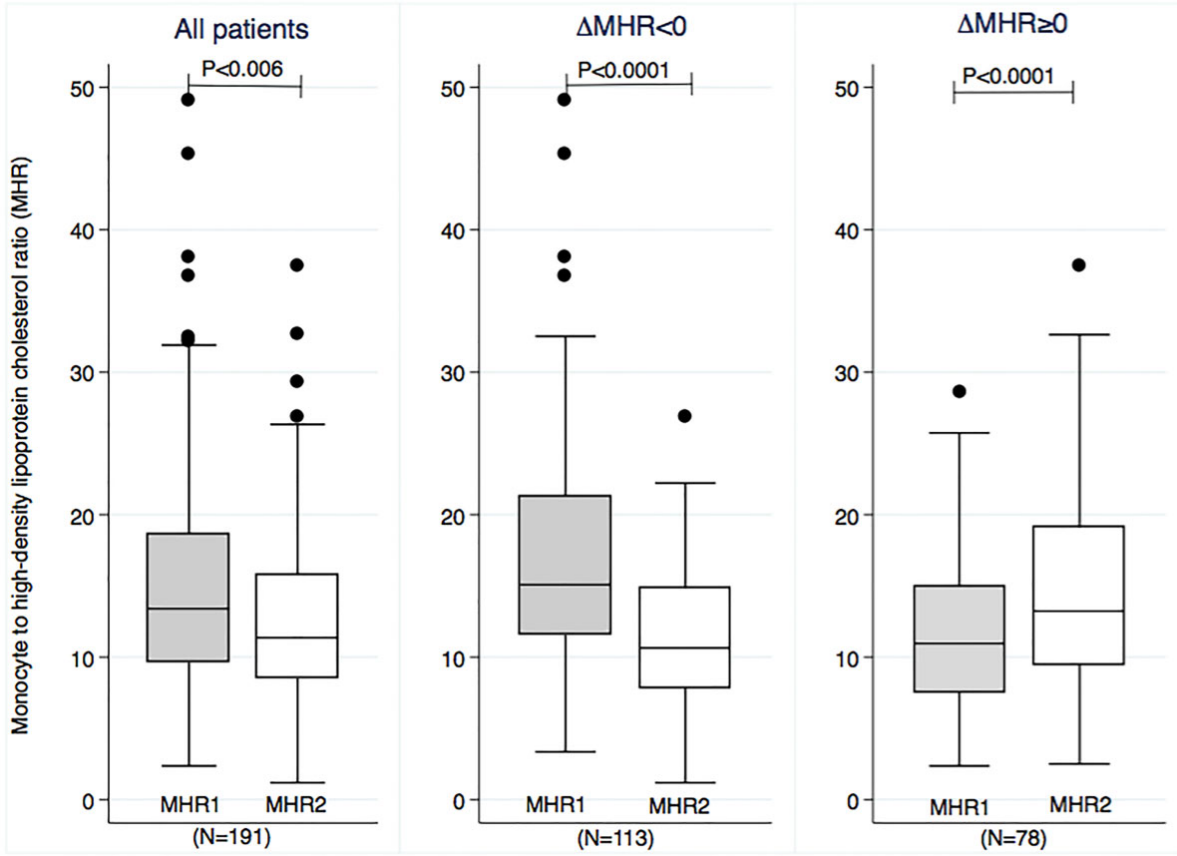
Box-plot graph comparing the MHR1 and MHR2 in all patients included in this study (left panel), in the patients whose MHR decreased over time (ΔMHR<0) (middle panel), and in the patients whose MHR increased over time (ΔMHR≥0) (right panel). Data are reported as medians and interquartile ranges; Wilcoxon matched-pair signed rank test. MHR1: monocyte to high-density lipoprotein cholesterol ratio at hospital admission; MHR2: monocyte to high-density lipoprotein cholesterol ratio at first outpatient evaluation; ΔMHR: MHR2-MHR1.

The time between the two MHR was similar in the groups, with 71 days (IQR: 50-93) in the ΔMHR<0 group and 66 days (IQR: 39-93) in the ΔMHR≥0 group (P=0.226). In general, the MHR decreased over time: 13 (IQR: 10-19) *vs* 11 (IQR: 9-16); P<0.006. The MHR reduction over time was the expected behavior for this marker. However, in a significant percentage of patients (78/191 [41%]), this ratio remained unaltered or rose over time (Figure 2).

As expected with high-potency statin treatment, LDL-cholesterol levels decreased over time: 100 mg/dL (IQR: 74-128) *vs* 79 mg/dL (63-94), P<0.0001; HDL-cholesterol levels increased slightly over time: 35 mg/dL (IQR: 30-40) *vs* 37 mg/dL (IQR: 32-43), P=0.050; and triglycerides levels were unchanged over time: 126 mg/dL (IQR: 93-179) *vs* 121 mg/dL (IQR: 92-168), P=0.077. Regarding monocyte count, we observed a decrease in monocyte numbers over time: 500 cells/mm^3^ (IQR: 400-600) *vs* 400 cells/mm^3^ (IQR: 300-600); P<0.0001.

**Figure 3:**
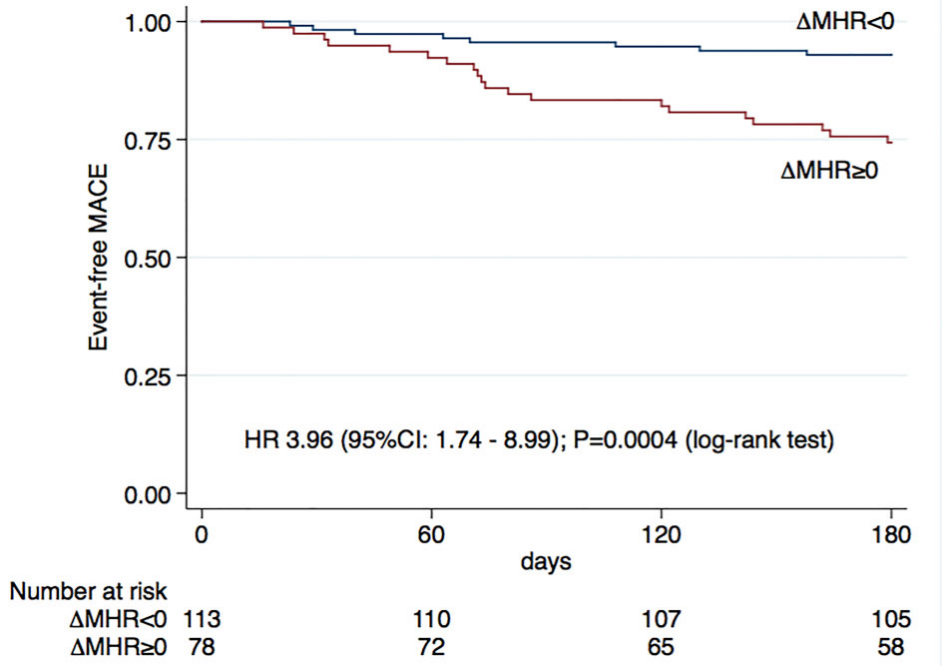
Kaplan-Meier curves showing the proportion of event-free major adverse cardiovascular events (MACE) according to patients’ ΔMHR. MHR1: monocyte to high-density lipoprotein cholesterol ratio at hospital admission; MHR2: monocyte to high-density lipoprotein cholesterol ratio at first outpatient evaluation; ΔMHR: MHR2-MHR1; HR: hazard ratio; CI: confidence interval.

During a mean follow-up of 166±38 days, we observed a higher occurrence of MACE in the ΔMHR≥0 (22%) than in the ΔMHR<0 group (7%), with a hazard ratio (HR) of 3.96 (95%CI: 1.74-8.99); P=0.0004 (Figure 3). The analysis of each isolated outcome is shown in Table 2. In the multivariable model, we included the following variables: gender, cardiac arrest on admission, Killip classification, left ventricle ejection fraction (LVEF), and GRACE score. After adjustment for these variables, the ΔMHR≥0 remained associated with a higher MACE occurrence, with an adjusted HR of 3.13 (95%CI: 1.35-7.26; P=0.008) (Table 3).

**Table 2:** Number of registered outcomes in ΔMHR<0 and ΔMHR≥0 groups.

Outcomes	ΔMHR<0 (n=113)	ΔMHR≥0 (n=78)	P
Primary outcome, n (%)			
MACE	8 (7)	17 (22)	0.003
Isolated outcome, n (%)			
Cardiovascular mortality	0 (0)	5 (6)	0.011
New AMI	4 (3)	2 (2)	1.000
Stroke	1 (0)	0 (0)	1.000
Hospitalization for heart failure	3 (2)	7 (8)	0.094
Unplanned coronary revascularization	0 (0)	3 (3)	0.066
Secondary outcome, n (%)			
All-cause mortality	0 (0)	8 (10)	0.0006

Chi-squared test was used for statistical analyses. MACE: major adverse cardiovascular events; AMI: acute myocardial infarction. MHR: monocyte to high-density lipoprotein cholesterol ratio.

**Table 3:** Univariate and multivariate analysis for major adverse cardiovascular event predictors.

Variables	Univariate			Multivariate		
	HR	95%CI	P	HR	95%CI	P
ΔMHR≥0	3.96	1.74-8.99	0.001	3.13	1.35-7.26	0.008
Male *vs* female	0.48	0.23-1.01	0.054	0.37	0.16-0.85	0.019
Age	1.01	0.98-1.04	0.473	-	-	-
ST-elevation AMI *vs* other types	0.87	0.41-1.85	0.726	-	-	-
Cardiac arrest on admission	2.79	0.97-8.06	0.057	1.82	0.48-6.91	0.380
Killip classification (II, III, IV *vs* I)	2.06	0.95-4.46	0.066	1.08	0.67-1.75	0.746
LVEF	0.96	0.93-0.99	0.008	0.97	0.94-1.01	0.110
GRACE	1.02	1.01-1.03	0.003	1.01	0.99-1.03	0.174
Coronary angioplasty	1.12	0.49-2.54	0.785	-	-	-
CABG	2.12	0.50-8.92	0.307	-	-	-
Diabetes	1.13	0.53-2.38	0.757	-	-	-
Number of affected vessels	1.34	0.87-2.08	0.189	-	-	-

Cox proportional hazard model was used for statistical analyses. We included in the multivariate model the variables that achieved a P-value <0.10 in the univariate analysis. HR: hazard ratio; CI: confidence interval; MHR1: monocyte to high-density lipoprotein cholesterol ratio during hospital admission; MHR2: monocyte to high-density lipoprotein cholesterol ratio during first outpatient evaluation; ΔMHR: MHR2-MHR1; LVEF: left ventricle ejection fraction; GRACE: global registry of acute coronary events; CABG: coronary artery bypass graft.

Through ROC-analysis, ΔMHR exhibited a superior prognostic accuracy to predict MACE [AUC of 0.73 (95%CI: 0.63-0.83)] compared to the MHR1 [AUC of 0.49 (95%CI: 0.38-0.60)] (P=0.004). Despite the higher AUC of ΔMHR, the difference from the MHR2 was not statistically significant [AUC of 0.65 (95%CI: 0.53-0.77)] (P=0.167). The MHR2 accuracy for MACE prediction was significantly greater compared to the MHR1 (P=0.0002).

## Discussion

This study showed that ΔMHR was a MACE predictor after ACS. To our knowledge, it was the first study to evaluate the ΔMHR in the context of all ACS types. Previous studies only assessed the isolated MHR during hospitalization mainly for ST-segment elevation AMI.

The MHR reduction over time is expected because more prominent inflammatory activity is observed during the acute phase (10,17,18), which plummets during the outpatient follow-up due to many factors, including the use of high-potency statins. However, as observed in our study, a meaningful percentage of patients (41%) did not exhibit a reduction of MHR over time, and this finding conferred a 3-fold increased risk of MACE occurrence during the 180-day prospective follow-up.

Avcı et al. (19) evaluated the MHR in patients admitted for ST-elevation AMI who underwent primary angioplasty and showed a sensitivity of 88.5% and a specificity of 49.5% (AUC of 0.756; P<0.01) for long-term MACE prediction and a sensitivity of 60.5% and a specificity of 65.6% (AUC 0.639; P<0.01) for in-hospital MACE prediction. Similarly, Açıkgöz et al. (20) evaluated the MHR as a predictor of cardiovascular events in patients with ST-segment elevation AMI. MHR was also an independent predictor for long-term mortality [HR 2.05 (95%CI: 1.23-4.09); P=0.014]. A higher MACE occurrence was observed in the in-hospital patients and in the 5-year follow-up with the highest MHR values. Çiçek et al. (21) showed that a higher MHR obtained during hospital admission was independently associated with increased in-hospital and long-term mortality and a more extended hospital stay in patients with ST-segment elevation AMI treated with primary angioplasty. The MHR was positively correlated with hsCRP and with the SYNTAX score. A recent meta-analysis evaluated the association between MHR and MACE among 2793 patients with ST-segment elevation AMI who underwent primary angioplasty. A higher MHR at hospital admission was associated with a significantly higher in-hospital mortality [relative risk (RR) 4.71 (95%CI: 2.36-9.39); P<0.00001] and the in-hospital MACE occurrence [RR 1.90 (95%CI: 1.44-2.50); P<0.00001], but this association was not observed for the long-term mortality (22).

MACE occurrence was statistically higher in the ΔMHR≥0 group. A statistically significant difference was observed for all-cause mortality and cardiovascular mortality when analyzing individual outcomes, but not for other isolated events. This finding could be attributed to insufficient sample size to assess these isolated outcomes.

The second MHR was obtained at the first outpatient evaluation in our study, approximately 66-71 days after hospital discharge. However, the most appropriate time for this second measurement has not been defined.

Other hematological indices have been suggested as cardiovascular predictors in ACS patients, particularly the neutrophil/lymphocyte ratio (NLR). A recent study in patients with ST-segment elevation AMI showed that the NLR was an in-hospital mortality predictor. However, after multivariable analysis using Cox regression, only age, left ventricle ejection fraction, and MHR were independently associated with long-term mortality (23).

### Clinical implications

The ΔMHR could select a subgroup of ACS patients with significant systemic inflammatory activation even after conventional drug treatment, which could benefit from other anti-inflammatory pharmacological interventions, such as monoclonal antibody anti-interleukin-1β (IL-1β). However, this use needs to be validated in clinical studies conducted for this purpose.

### Limitations

This was a single-center study with a small number of patients, and these results need to be reproduced in prospective cohorts with a higher number of patients from other centers. The ΔMHR did not identify early cardiovascular events occurring between hospital discharge and the first outpatient evaluation; therefore, new studies are needed to evaluate if this time interval until the second measurement could be shorted. ΔMHR showed a moderate accuracy in determining MACE occurrence; because of this, ΔMHR should be used together with other predictive biomarkers in clinical practice. Because of the number of events (38/191 (20%)) and the large 95%CI in multivariable analysis, we cannot exclude that there was overfitting in this model. We did not measure other inflammatory markers, such as hsCRP and IL-1β, to correlate their circulating levels with ΔMHR. Future studies need to assess whether ΔMHR has an incremental effect on inflammatory markers already used in clinical practice, such as hsCRP.

### Conclusion

The ΔMHR was an independent MACE predictor after ACS and is a potential marker of residual cardiovascular risk after ACS.
